# Investigations of the Polycyclic Aromatic Hydrocarbon and Elemental Profile of Smoked Fish

**DOI:** 10.3390/molecules27207015

**Published:** 2022-10-18

**Authors:** Elif Tuğçe Aksun Tümerkan

**Affiliations:** 1 Department of Food Processing-Food Technology, Vocational School of Health Services, Ankara Yıldırım Beyazıt University, Ankara 06010, Turkey; etaksun.tumerkan@aybu.edu.tr; 2 AYBU Central Research Laboratory, Application and Research Center, Ankara Yıldırım Beyazıt University, Ankara 06010, Turkey

**Keywords:** bioaccumulation, heavy metal, polycyclic aromatic hydrocarbons, processing yield, smoking, water holding capacity

## Abstract

Fish are vulnerable to environmental pollutants such as polycyclic aromatic hydrocarbon and heavy metals. As one of the most commonly applied processing methods, the smoking of different species has been applied globally. Hence, this study aims to investigate the smoking process on the polycyclic aromatic hydrocarbon and elemental accumulation of the five different species (rainbow trout, Atlantic bonito, horse mackerel, sea bass, and Atlantic bluefin tuna) which are commonly processed and traded in the smoked fish industry. The processing yield, water holding capacity, and pH were also investigated. The results revealed that the proximal differences among fish species influence the water holding capacity, processing yield, and pH which are very important for process sustainability and the quality of the end product. The main finding was the proximal composition impact on the accumulation of both PAHs and heavy metals at different levels. While all of the tested samples were below the maximum permissible limit, some of the heavy metals, especially toxic elements, were found above the acceptable limit. Horse mackerel is determined to be the species most vulnerable to PAHs and heavy metal accumulation.

## 1. Introduction

With the increasing population and food security concerns, the importance of fish in human nutrition has been well understood around the world in recent years. Fish consumption (150 g) meets approximately 60% of the daily protein needs of adults [1]. Fish consumption is considered to be the main source of many nutrients for human health, from vitamins to fatty acids and minerals [2]. While fish has historically been consumed fresh, various processing techniques have begun to be applied depending on the changing consumer demand and technological developments. Several processing techniques are applied to extend the shelf life of fish or to improve the organoleptic properties of fish [3,4]. Smoking is known as one of the oldest traditional processing techniques that comprises salting, drying, and thermal treatment. In addition to other processing methods, several alternative products are offered to the consumers by improving the taste and aroma of sawdust through the volatile compounds released from the wood or sawdust in the smoking process [5]. Despite these benefits, as an undesired result of smoking, toxic chemicals such as polycyclic aromatic hydrocarbons (PAH), nitrosamines, heterocyclic amines, and heavy metals are generated due to the pyrolysis of wood lignin and organic compounds within thermal processes [6]. Harmful chemicals, especially PAHs and heavy metals, that are formed or accelerated during the smoking process threaten human health through the consumption of smoked food products [7]. Different committees around the world, from the Joint Food and Agriculture Organization of the United Nations and the World Health Organization Expert Committee on Food Additives (JECFA FAO-WHO) to the European Food Safety Authority (EFSA) classified PAHs and heavy metals as some of the most dangerous contaminants due to their genotoxic, carcinogenic and mutagenic properties, specifically in regard to human health [8].

Among the 24 described PAHs, benzo[a]pyrene (BaP) was accepted as carcinogenic and the other 3 PAHs, benzo(b) fluoranthene (BbF), chrysene (CHR), benzo(a)anthracene (BaA), were categorized as having a high potential to be carcinogenic to humans, and these four most risky components are grouped as PAH4 [9]. The European Commission has strictly set the maximum limit as 2 μg/kg for BaP and 12 μg/kg for PAH4 (the sum of BaP, CHR, BaA, and BbF) in smoked fish products [10]. These lipophilic contaminants are also classified into two categories according to the number of aromatic rings they contain and their molecular weight; while the structure of PAH contained two to three aromatic rings were considered to be low molecular weight compounds (LPAHs), high molecular weight compounds (HPAHs) are present when four and more aromatic rings are formed in the structure [11]. The heaviest PAHs tend to be riskier for humans due to not only having high stability and capacity for toxicity, but also with the greatest lipophilic properties which increases the concern in the food industry [12,13]. Heavy metals are another significant risk for smoked fish, and are considered a food security concern. Especially, lead (Pb), cadmium (Cd), mercury (Hg), and arsenic (As) show toxic effects, even at low concentrations [14]. The potential risks of PAHs and heavy metals are relatively higher in fish than in other food items due to these pollutants already being present in the water bodies where the fish live, and they can be bioaccumulated in the fish [15,16]. The bioaccumulation or formation of these pollutants in processed fish can be affected by internal factors, such as the proximate composition and pH of the fish, external factors such as processing time and temperature applied, and distance between the fish surface and heat sources [17]. Alongside other smoked products, PAHs, and heavy metals in smoked fish have been subjected to a wide range of research globally as a part of food safety concerns.

There is some research conducted on the determination of PAHs or heavy metal contamination in commercial fish products, separately from Turkey [18,19,20], however to our knowledge, no studies have been conducted to evaluate the smoking process together on heavy metal and PAH formation in various fish. There is a need to study the risk assessment in the most commonly smoked fish species in Turkey. Trout, mackerel, bonito, sea bass, and tuna are among the most consumed species in Turkey, and the smoked forms of these species have a market size in both national and international seafood markets. Therefore, a preliminary study was conducted to holistically evaluate the effects of hot smoking on the formation of PAHs and the elemental profile of these five species by addressing the physicochemical properties of fish.

## 2. Materials and Methods

### 2.1. Materials

The study material comprised the five fish species of trout, Atlantic bonito, sea bass, and Atlantic bluefin tuna obtained from local fishermen from different cities in Turkey in early 2022. Atlantic bluefin tuna (*T. thynnus*), from İzmir, sea bass from Bodrum, trout from Antalya, Atlantic bonito from Trabzon and horse mackerel originated from Iceland obtained from an international seafood processing plant in Antalya. Within one species, the fish were chosen with uniform size and weight. The variations in the size between individual fish did not exceed 5% of the mean value that represented 30 fish presented at the fishermen’s place. Following harvesting, the fresh fish was divided into two equal batches. Samples of 15 of the individual fresh fish were transferred under a cold chain to Ankara Yıldırım Beyazıt university laboratory immediately. Fish were descaled, eviscerated, cleaned, minced, homogenized and packed in amber bottles and kept in the freezer until the following fresh analysis. The remaining fish samples from each species (15 individuals of each fish) were transferred under a cold chain to the seafood processing plant immediately.

### 2.2. Smoking Process

Hot smoking was carried out at the commercial seafood processing plant in Antalya, Turkey. The same smoking process was applied to all fish species to avoid variations in the results that could be caused by differences in the smoking process. The fish were eviscerated, and washed with the cold water thoroughly, the fish were then dipped into a brine solution (7.5% of salt) for 8 h and drained. The cured fish were washed again through cold water and then dried at 9–10 °C for 1.5 h with high air circulation. Brined and dried fish were then placed into a professional smoking chamber Air Master UK-1800 BE (Reich, Germany) and smoked by direct smoking with smoke produced from oak wood at 100–120 °C for 2 h until reaching the internal temperature of fish 90 °C which was controlled by Fisher brand traceable alarm thermometer/timer (Fisher Scientific, Pittsburgh, PA). Then, the smoked fish were filleted and de-boned in the cold room (10–12 °C). The smoked fish fillets were transferred to the laboratory under the cold chain and then minced, homogenized and packed in amber bottles and kept in the freezer until the following analysis.

### 2.3. Proximate Composition of Fish

The proximate composition of the fresh spotted fish and smoked spotted fish from each species were determined by the following methods: crude protein content was determined according to the Kjeldahl method [21]. One g of raw and smoked fish material was hydrolyzed with 25 mL concentrated sulfuric acid (H2SO4) containing two catalyst tablets in a heat block (Büchi Digestion Unit K-424, Labortechnik AG, Flawil, Switzerland) at 370 °C for 2 h. Hydrolysates were then cooled at room temperature, H2O was added to the before neutralization and titration, using a Büchi Distillation Unit B-324 (Büchi Labortechnik AG, Switzerland). The crude protein level was determined by protein conversion factor 6.25 (N × 6.25). The total lipid content of the fish sample was analyzed following the method of Bligh and Dyer [22] with the aim for a single-phase lipid solubilization to be used. Approximately 10 g of the fish sample was homogenized in the chloroform-methanol mixture (1:2) for 1 min while being held in ice/water. Then, 20 mL chloroform was added to the mixture and then homogenized again for 30 s. The homogenate was transferred into centrifuge tubes and centrifuged for 20 min at 2000 rpm. The lower chloroform phase was removed and then 20 mL of a chloroform solution containing lipid was pipetted into another flask. The solvent was then evaporated using a rotary evaporator under vacuum (Büchi Labortechnik, Flawil, Switzerland) and finally then placed into the oven at 60 °C for 1 h. Total lipid content was calculated gravimetrically, by evaporating a defined amount of extract. The moisture content of smoked fish was analyzed following the AOAC method [23]. Five g of each raw and smoked fish were placed in an oven (Memmert GmbH + Co, Schwabach, Germany) and dried overnight at 103 °C ± 2. Dried samples were transferred to desiccator to cool, reweighed and the content of moisture calculated. The ash content of fresh and smoked fish samples (4–5 g of fish) was analyzed by incineration procedures in electric furnaces at 550 °C (AOAC) [23]. Fish samples were then cooled in the desiccator and weighted again and calculated gravimetrically.

### 2.4. Determination of Water Holding Capacity, Process Yield and pH Measurement

A Weilheim pH meter (Weilheim, Germany, model 15i/SET) was equipped with a glass electrode that was used to measure the pH of the fish flesh following the calibration of the pH meter using pH 4.0 and pH 7.0 buffers. Fish samples were homogenized in distilled water (1:10 *w*/*v*) with a high-speed homogenizer in an ice bath at 12,000 rpm for 3 min (Ika-Werke Ultra-turrax, IKA Instruments Ltd., Schwabach, Germany) as described by Santos [24]. The water holding capacity was determined using the method described by Messina et al. [25] with some modifications. Briefly, homogenized fish muscle (2 g; Ws) was placed in a centrifuge tube along with weighted filter paper (Wi) and centrifuged at 3000× *g* for 10 min at 20 °C. Following the centrifugation, the fish sample was removed and the filter paper again weighted (Wf). Water holding capacity (expressed as %) was calculated as the difference between the percentage of the initial water content in the muscle and the water released after the centrifuge. The samples were analyzed in triplicate. The processing yield was determined according to Zhou et al. [26]. The processing yield was calculated as the ratio of the weight of each fillet before salting to its weight after the smoking process, and was expressed as a percentage.

### 2.5. Determination of Polycyclic Aromatic Hydrocarbons (PAH)s

With the aim to determine the smoking processes impact on the PAHs formation in the fish muscle, PAHs were analyzed from both unsmoked and smoked samples from each species according to the method described by Pule et al. [27]. PAHs compounds were extracted using the QuEChERS methods; 5 g of fish samples and 10 mL of HPLC grade acetonitrile were vortexed for 1 min. Magnesium sulfate (MgSO4) and sodium acetate (NaOAc) were then added to mixture, vortexed for 1 min and centrifuged at 4000 rpm for 5 min. The upper phase was transferred into an AOAC QuEChERS dispersive solid phase extraction tubes which contains 300 mg of primary secondary amines, 30 mg of C18 EC and 900 mg of anhydrous MgSO4. The mixture was vortexed for 1 min and then centrifuged at 4000 rpm for 5 min. The extract was filtered through a 0.20 mm polyvinylidene fluoride syringe filter and then 0.1 mL of the extract was analyzed by HPLC coupled with Shimadzu 10AxL fluorescence detector (Excitation: 254 nm, Emission: 390) with Phenomenex HyperClone BDS C18 Column. The mobile phase composition was Pump A (Acetonitrile) and Pump B (Deionized Water) at 0.8 mL/min.

All the reagents used in physicochemical analyses, and PAHs analyses were obtained from Merck, Sigma, etc. Methanolic 2 M-KOH (methanol/water 9 + 1) and hexane analytical grade were redistilled in the glass before use. Methanol (analytical grade), Silica gel (mesh: 70–230), glass wool and potassium hydroxide pellets (Purity: 86.1%) were obtained from Sigma Aldrich. The PAH standard mixture contained 16 PAHs compounds (purity: 95.9–99.9%). The PAHs in fish samples were identified using the retention times against the standards and quantified using the calibration curve develop; the concentration of the PAH in the standard mix was as follows: 2.5 mg kg1 for Naph, 1 MN, 2 MN, and Ace; 5 mg kg1 for Ant, Pyr, Flu, BbF, and BkF; 10 mg kg1 for Flu and BaP then 20 mg kg1 for DahA. The sum of PAH4 including the sum of actual values for benzo[a]pyrene, benz[a]anthracene, chrysene and benzo[b]fluoranthene is reported.

Analysis of procedural (reagent) blanks, field blanks and recovery tests were used to check for contamination and performance of the method. The recovery tests were performed at concentrations of PAHs ranging from 0.05 to 0.8 μg/mL. No significant peaks appeared in the chromatograms of the blanks. Fish samples were also used for recovery determination by spiking of the muscles with 500 ng/g of the PAHs mixture. The average recovery rates were ranged between 85.40% and 114.20%. The detection limit was defined as concentration corresponding to three times the noise value measured. The limit of detection (LOD) of each PAHs was determined based on the standard deviation of the response to the slope of the calibration curves R values, and recovery rates are given in Supplementary Materials Table S1.

### 2.6. Elemental Profile Analysis

The heavy metal analysis on fresh fish and smoked fish was determined by the AOAC International (2000) method using an atomic absorption spectrophotometer. All chemicals used in this study were of analytical grade. Fish samples were transferred to the Science and Technology Application and Research Center (STARC) Yozgat Bozok University for analysis in a cold chain setting. The samples were pretreated before analysis at this center as follows; 3 g of each sample was dried at 37 °C for 24 h, and then 0.3 g of each sample placed into polypropylene tube and then 5 mL of Suprapur nitric acid (HNO_3_) and 2 mL of peroxide (H_2_O_2_) were mixed and left at the room temperature to allow for the dissolving process to take place. The mixture was then diluted with ultra-pure water (10 mL). The analysis of heavy metals and trace elements was performed in an inductively coupled plasma mass spectrometer (ICP, Thermo Scientific ICAP Qc, Omaha, NE, USA). An 11-point calibration curve was generated for each element, and internationally validated certified standard samples. Certified reference materials, CRMs ERM^®^-BB422 (fish muscle), was acquired from the Institute for Reference Materials and Measurement of the Joint Research Centre (JRC) of the European Commission (Geel, Belgium) were used for the validation of these techniques. Nitric acid (SuprapurVR, 65%) was used for the digestion of samples and standard reference material. Ultra-pure water (Direct-QVR; Millipore, Darmstadt, Germany) was used for dilution of the standard (multi-element standard ChemLab, Zedelgem, Belgium) and sample preparations.

The sixteen metals (cadmium, lead, and vanadium) were measured using inductively coupled plasma mass spectrometry (Thermo Scientific ICAPQc, Omaha, NE, USA). The operating parameters were set as follows: radiofrequency power 1550 W, nebulizer pressure 3.01 bar, plasma gas 0.80 L/min nebulizer gas 3.04 L/min, dwell time 0.01 milliseconds, and spray chamber temperature 2.9 °C. The sampler probe was washed between injections by rinsing with ultra-pure water for 45 s, followed by washing with 2% HNO_3_ for 45 s, and finally rinsing with ultra-pure water for 45 s. The instrument was operated in the quantitative mode (linear calibration; R^2^ > 0.99) and the interval of calibration was set at 0.1–500 μg/L for all elements. Certified Reference Material (CRM-SeronormTM Trace Elements Whole Blood L-2, Sero AS, Billingstad, Norway) was used for the validation method. A mixture of internal standards (Hf) was used to check the stability and sensitivity of the instrument. The limit of detection (LOD) of each element was determined based on the standard deviation of the response to the slope of the calibration curves. (available in Supplementary Materilas Table S2). The samples of heavy metals were performed in five replications.

### 2.7. Health Risk Assessment of PAHs and Trace Elements

Health risks of PAHs and trace elements were assessed by daily dietary intake (DDI) and the health implications associated with the PAHs in fish were assessed by calculating the carcinogenic potencies of individual PAHs.

The daily dietary intake (DDI) was calculated with Equation (1),

DDI = C × IFR(1)

where C is concentration of PAHs (single or sum) in fish tissue and IFR (g day^−1^) is the fish ingestion rate. Fish ingestion rate is 17.29 g person/day in Turkey [28].

Carcinogenic potencies of individual PAHs(B(A)Pteq) were determined by Formula (3), where Ci (mg/kg) is the concentration of a single PAH (i) compound in fish tissue and TEF sign as the toxicity equivalence factor of the relevant components. As an accepted formally toxic equivalency factors (TEF) values were obtained from Nisbet and Lagoy [29].

(B (A) Pteq) = Ci × TEFi B (A) Peq = ∑Ci × TEFi(2)

For the health risk assessment of the most dangerous trace elements, the estimation of daily intake rate (EDI) and target hazard quotient (THQ) values were determined according to the following formula;

EDI (μg tested element kg/body weight/day).

(C _element_ × D _food intake_) (3)

B_average weight_;

C_element_: the average element concentration in fish;

D_food intake_: daily fish consumption rate (kg/person).

In this health risk assessment, the fish ingestion rate is 17.29 g person/day in Turkey [28] and 70 kg of body weight refers to the optimal value for adult humans [30]. THQ values determined for the assessment of the cancerogenic risks can be obtained by consumption of the tested fish samples. THQ was calculated with the formula according to formula ([31]),

(4) $$\mathrm{THQ}=\frac{E_{\mathrm{fr}}{\times \mathrm{ED}}_{\mathrm{tot}}\times \mathrm{FIR}\times C\;}{{\mathrm{RfD}}_{o}{\times \mathrm{BW}}_{a}{\times \mathrm{AT}}_{n}}{\times 10}^{-3}$$

E_fr_: frequency of exposure (365 days/year)

ED_tot_: period of exposure (average life expectancy: 70 years)

FIR: pood intake rate (0.019 g for per day)

C: mean of detected heavy metal concentration in fish muscular tissue (mg/kg)

* RfD_o_: oral reference dose (mg/kg/day)

BW_a_: average body weight (For adult people,70 kg refers to body weight)

AT_n_: period of average exposure for non-carcinogens (365 days year−1 × number of exposure years)

* oral reference doses (RfD_o_) for each metal were declared by the US EPA [32] (Cr: 1.5, As: 3 × 10 − 4, Cd: 1 × 10 − 3, Al: 1.0, Cu: 0.04, Ni: 0.02, Hg: 3 × 10 − 4, Pb: 4 × 10 − 3.)

### 2.8. Statistical Analysis

All analyses were carried out in triplicate (for elemental profile *n* = 5) and the results are given as average ± standard deviation. The data was analyzed using SPSS 22.0 software (IBM Corp., released 2011. IBM SPSS Statistics for Windows, version 20.0, Armonk, NY: IBM Corp. IBM SPSS Statistics for Windows, version 20.0, Armonk, NY, USA) Categorical data were compared with the chi-square test. The results were considered statistically significant at *p* < 0.05. Pearson correlation coefficients for the relationships between the PAHs compound and proximate composition parameters (*n* = 5) in the fish muscle were determined using SPSS version 20 (*p* < 0.05).

## 3. Results and Discussion

### 3.1. Proximate Composition Differences among Raw and Smoked Fish Samples

The average contents of water, ash, crude fat, and protein of fish before and after the smoking process are presented in Table 1. The proximate composition of fish species differs from species to species.

The nutritional value of the fish products is driven not only by type of fish, but also by the type of processing method. In addition to the processing time and temperature effects on the nutritional value of processed fish, food matrices during processing, such as salt, oil or any type of additives, also cause variation in the proximate composition of the end products [33]. Due to smoking process includes brining, drying and smoking applications, the differences in protein and lipid levels of raw and smoked fish species are generally high [34]. The variations in proximal compositions of fish species after smoking process are shown in Table 1. In the present study, there was a significant increase detected in the protein level among fresh and smoked fish, regardless of the kind or species. On the contrary, the total lipid and moisture levels decreased throughout the smoking process in all fish species, which can be explained by fish physically losing fat and moisture during the smoking process [34,35].

Table 1 shows the moisture, ash, lipid, and protein contents for each fresh and smoked fish species. Statically significant differences were found in moisture, lipid, and protein contents between species. It is well known that the proximate composition of fish muscle is driven by several factors, such as inherent genetic properties, seasonal differences, and environmental conditions. The highest and the lowest crude protein rates were determined in raw trout (22.9%) and Atlantic bonito (14.3%), respectively. The lipid content of fish species determined in an order from highest to lowest is as follows: horse mackerel (25.4%) > Atlantic bonito (17.8%) > Atlantic bluefin tuna > (10.7%) trout (4.5%) > sea bass (4.20%). The protein and lipid contents were determined as 19.8% and 4.2% in sea bass, respectively. These results coincide with the findings of previous research which reported that the protein and lipid contents were 19.36% and 5.14%, respectively, in sea bass (Yazgan et al. [36]). Proximate composition (crude protein, lipid, moisture, and ash) of mackerel was determined to 15. 7%, 25.4%, 53.4% and 1.5%, respectively, which is consistent with the findings reported by Malesa-Ciećwierz et al. [37] who stated that the composition was as follows: moisture (54.7%, total lipid (26.8%, crude protein 16.8% and ash 1.7%.

Since tuna is an umbrella species, a proximate composite of sub-tuna species differs from each other. In this study, the proximate composition of Atlantic bluefin tuna (T. thynnus) was determined as moisture (63.9%), total lipid (10.7%), crude protein (21.9%), and ash (2.1%) which is in accordance with previous research that indicated that the proximate composition of farmed and wild Atlantic bluefin tuna consisted of the following: lipid rate (11.04–12.85%), protein rate (20.96–21.09%) and moisture rate (61.03–63.28%) [26]. The proximal values of Atlantic bonito were determined as: protein (14.3%), fat (17.8%), moisture (68.2%), and ash (1.8%) (Table 1), which were in agreement with results reported by Koral et al. [38] who indicated the protein, lipid and moisture levels as 14.55, 12.87 and 67.71%, respectively. As one of the most commonly used freshwater fish species, a wide range of research conducted on the proximate composition of trout varies depending on feeding, season, and gender. The protein and total lipid rates of rainbow trout were found as 22.9% and 4.5%, respectively, in this study which agrees with the findings of Karimian et al. [39] who reported the crude protein as 18.59% and total lipid level as 3.58% for rainbow trout.

### 3.2. pH, Processing Yield and Water Holding Capacity

pH value is accepted as another parameter which impacts the other physiochemical, microbiological and sensory quality attributes of the processed fish. In this study, pH value significantly (*p* < 0.05) decreased after the smoking process regardless of the type of fish species (Table 2). While the pH value decreased from 5.8 to 5.3 in rainbow trout following the smoking process, the pH found in fresh mackerel was 6.4, and this value reduced to 6.1. The same decline in pH value was observed in Atlantic bonito, from 5.8 to 5.4 in fresh and smoked form, respectively. The highest pH value detected in Atlantic bluefin tuna was 6.8 among the fresh fish species, and this value decreased to 6.3 in the smoked form. A slight pH decline was observed in sea bass before and after the smoking process (from 6.2 to 6.0, respectively). A similar decline in pH was reported from 6.33 to 6.04 for sea bass by Fuestes et al. [40]. The pH decline driven by the smoking process was also reported by Çoban et al. [41]. They stated that the pH was 6.48 for raw trout and this value reduced to 5.75 following the smoking process.

WHC is regarded as one of the important parameters for the texture and general appearance of processed fish products. In this research, the WHC increased after the smoking process, regardless of fish species (*p* < 0.05). The lowest and highest WHC in raw fish fillets were found in bluefin tuna and Atlantic bonito, at 81.45% and 89.54%, respectively. A relatively bigger increase in WHC was observed between fresh and smoked sea bass from 83.4 to 93.4%, respectively, among the tested groups, which is in the accordance with the previous findings by Fuentes et al. [40] who stated that the WHC rose from 77% to 88% in fresh and smoked sea bass. The increased WHC can result in the synergistic effect of the salting and drying steps in the smoking process. Messina et al. [25] also reported that the WHC of meagre (*Argyrosomus regius*) fillets increased significantly following the smoking process.

Processing yield is one of the main concerns in profitability and economic achievement for sustainable seafood processing. The processing yield of smoked fish depends on both raw material characteristics, such as size and proximate composition and processing length and the optimization of the filleting steps and brine concentration [42]. The yield of smoking processes was found as 71.4% for rainbow trout, 90.2% for horse mackerel, 88.5% for Atlantic bonito, 72.1% for sea bass, and 84.6% for Atlantic bluefin tuna. These results were in agreement with previous research indicating that the processing yield in horse mackerel was between 77.7% and 81%, and 72.8 and 75.9% in Atlantic mackerel [43]. Similarly, Lerfall, and Hoel [44] reported that the processing yield differed from 88.4% and 98.4% in the smoked salmon range. In this study, among the five species smoked and analyzed within the same protocol, the higher processing yields were detected in the species, which have relatively higher levels of lipid. The correlation between the total fat content of fish species and processing yield was also highlighted by Cardinal et al. [42] who stated that fish species with less than 10% lipid content are more sensitive to processing conditions, and the processing yield is relatively lower than that of fatty fish.

### 3.3. The Concentrations of Polycyclic Aromatic Hydrocarbons (PAHS) in Raw and Smoked Fish

PAH content in raw and smoked fish species is summarized in Table 3. The presence of sixteen tested PAHs were detected at varying concentrations in the smoked fish samples. Most of the PAH contents were detected in raw fish species only in trace amounts. The content of PAH was increased following the smoking process in all fish species. In this study, the presence and formation of PAHs showed significant differences among the tested fish species. Both smoked and raw fish were dominated by benzo(a)anthracene (BaA), which is known as a carcinogenic and genotoxic PAH. The highest mean contents of BaA of all the raw samples were noted in horse mackerel at 1.19 μg/kg and in sea bass at 1.08 μg/kg, respectively. The mean content of BaA in the smoked fish were detected as follows: Atlantic bonito > horse mackerel > bluefin tuna > herring > rainbow trout = sea bass at 6.08 μg/kg, 2.56 μg/kg, 1.26 μg/kg, 1.19 μg/kg and 1.13 μg/kg, respectively. Benz(a)anthracene was higher in all raw fish samples than in the smoked samples, which means that the smoking process may have increased them. A relatively higher level of BaA was detected in the horse mackerel and bluefin tuna, which have higher fat contents. The formation of benzo[a]anthracene, impacted by the fat ratio of food items, was also highlighted by Kaplan İnce and İnce., [45]. Due to BaA having a high molecular weight, migration of this PAH to fish can occur easily and it is categorized in genotoxic PAHs; the controlling of BaA is important in fish products.

**Figure 1:**
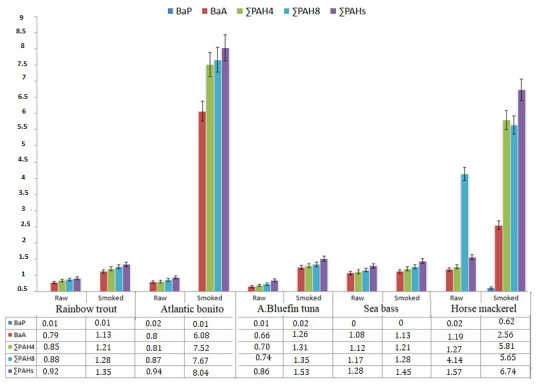
Most important PAHs contents in raw and smoked fish samples.

While the highest level of BaP was determined in the smoked horse mackerel, the BaP level was found to be at a trace level in smoked rainbow trout, smoked Atlantic bonito, and bluefin tuna. As in the fresh form, BaP was not detected in the smoked form of sea bass which is in accordance with Duedahl-Olesen et al. [46] who reported that BaP was not found in smoked fish samples. The major health concerns in the smoked food products are that the PAH4 content (the sum of B[a]P, benzo[a]anthracene-B[a]A, chrysene-Chr, benzo[b]fluoranthene) have been mutagenic and carcinogenic due to these PAHs [47]. In this study, relatively higher ∑PAH4 contents were determined in smoked Atlantic bonito and smoked horse mackerel as 7.52 and 5.81µg/kg, respectively (Figure 1). The ∑PAH4 level was detected as 1.21 µg/kg in both smoked rainbow trout and smoked sea bass, and 1.31 µg/kg in smoked Atlantic bluefin tuna. The levels of BaP and PAH4 did not exceed the maximum acceptable limit of 2 μg/kg and 12 μg/kg, respectively approved by both the European Commission (European Union with EC Regulation (EU) No 835/2011) and Turkish Food codex (TFC., 2011) in any of the smoked groups.

As seen in the Figure 1, the total genotoxic ∑PAH8s (the sum of benz[a]anthracene, chrysene, benzo[b]fluoranthene, benzo[k]fluoranthene, benzo[a]pyrene, indeno [1,2,3-cd]pyrene, dibenz[a,h]anthracene, and benzo [ghi]perylene) levels were found to differ significantly among the smoked fish groups. As EFSA indicated, BaP alone is not a good marker for the determination of the carcinogenic PAHs occurrence in the food products [47]; detection of PAH8 level is important for public health. Of the five smoked fish groups, except smoked horse mackerel, benz[a]anthracene was found to be the predominant PAHs which was followed by chrysene. The remaining genotoxic PAH8 was found in trace amounts in the relevant smoked fish samples. The dominance of PAHs was detected as benz[a]anthracene (2.56 µg/kg), benzo(b)fluoranthene (1.57 µg/kg) and followed by chrysene (1.06 µg/kg) in the smoked horse mackerel.

In order to avoiding any deviations from the process, the smoking process and all the applied analyzes were performed under the same conditions and following the same steps. Therefore, the relative variation in the PAH4 and PAH8 formation can be attributed to the fact that the difference of the proximal composition of the tested fish species, in particular total fat content. Since raw and smoked horse mackerel have the highest fat level in all tested sample groups, the composition of PAH4 and PAH8 can correlated to the relevant fat level. The higher lipid content in horse mackerel has facilitated the PAHs formation and bioaccumulation at higher levels. This correlation can be related to the lyophilic character of PAHs which is accepted as the main reason for the accumulation mechanism in lipids [48]. The relation between PAH composition of smoked fish was also proven by several researchers. Adeyeye and Ashaolu [49] highlighted that the PAH compounds formed by fat pyrosis in the smoked fish and the higher lipid content of fish resulted in higher PAH composition.

The ∑PAHs levels increased in all type of fish species after the smoking process, which means the smoking process led to PAHs formation, but at different levels. Nearly all (other than benzo[k]fluoranthene and phenanthrene) 16 PAHs were detected in only horse mackerel among the raw fish species (Figure 1). These variations in the composition of PAHs can be the result of the difference in the origin of the tested fish sample. In this study, except the horse mackerel, which originated from Iceland, all of the fish samples originated from Turkey. It is accepted that the distribution of PAHs in various water bodies is impacted by the seasonal and location differences and results in the bioaccumulation of PAHs different levels [50]. The higher ∑PAHs levels were determined as 8.04 and 6.74 µg/kg in smoked Atlantic bonito and smoked horse mackerel, respectively (Figure 1). While the ∑PAHs found at the highest level in smoked Atlantic bonito, the composition of PAHS, (16 of the tested PAHs) was found to be significantly different in the smoked horse mackerel when compared with the other smoked fish species. The present study revealed a strong correlation between the fat content of fish species and PAHs formation after smoking process. The PAH levels in all raw and smoked fish samples were below the maximum limit of 30 mg/kg as settled by the Turkish food codex.

### 3.4. Elemental Profile of Raw and Smoked Fish Samples

**Figure 2:**
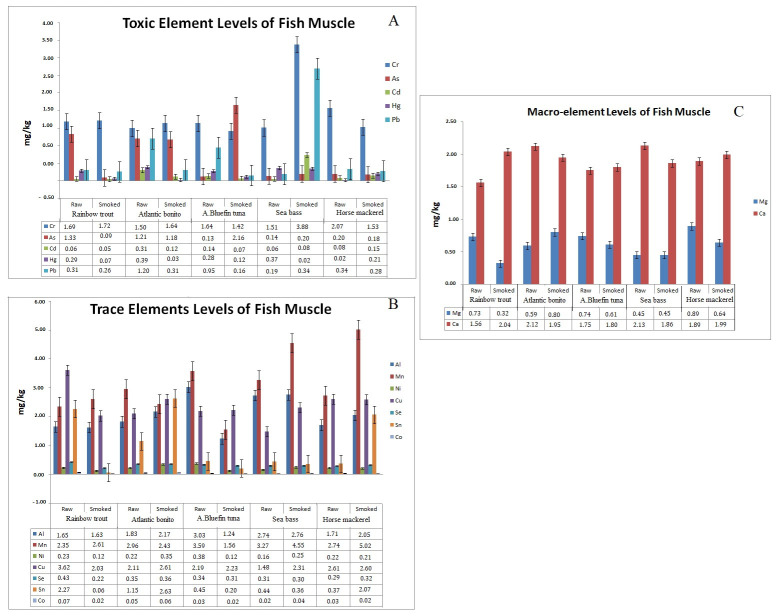
Elemental composition of fresh and smoked sample (**A**): toxic element level of both fresh and smoked samples, (**B**): trace element level of both fresh and smoked sample, (**C**): macro-element level of both fresh and smoked samples.

The heavy metal profile determined as the concentrations of sixteen elements (Al, Cr, Mn, Co, Ni, Cu, Zn, As, Se, Fe, Cd, Sn, Mg, Hg, Pb and Ca) before and after the smoking process in the different species are presented in Table 4. As shown in Table 4 and Figure 2A, there were significant differences in the amount of potentially toxic metals (Cd, Cr, Pb, Hg and As). When compared to the accumulation of toxic elements in the raw form of different species, there were significant differences detected. The quantity relationship between toxic elements in rainbow trout muscle was determined to be Cr >As > Pb > Hg > Cd. The toxic element levels from the highest to lowest level in order was determined to be Cr > Pb > As >Hg > Cd in horse mackerel and sea bass. This order was found to be Cr > As > Pb >Hg > Cd in Atlantic bonito, and Cr > Pb > Hg > Cd > As in bluefin tuna. These differences in the toxic metals among unprocessed fish species may be due to the origin of the species, environmental conditions, feeding and therefore the proximate composition. These results clearly indicate that each fish has a different capacity for bioaccumulation of toxic metals. Toxic element variations in different species were also the subjected of several studies; by Töre et al. [51], Djedjibegovic et al. [52] who reported that the toxic metals levels vary in the different fish species from Turkey and Bosnia and Herzegovina, respectively. There are many external and internal factors which affect the accumulation of toxic metals in fish muscle, from pH to salinity [53]. Several processing methods which comprise heating, salting or drying make fish muscle more vulnerable to the bioaccumulation of these metals on fish products. There were significant variations of raw and smoked fish samples also observed (Table 4 and Figure 2A). Except horse mackerel, the level of Hg was decreased in the remaining species after the smoking process, and the highest Hg level determined in smoked horse mackerel which has the lowest moisture content among smoked samples. These findings clearly revealed that the relationship between Hg and moisture level of fish is in accordance with a previous study, highlighted by the reduction in Hg concentrations driven by thermal processes and moisture content [54].

Except sea bass, the Pb levels were decreased in all remaining fish samples after the smoking process. The higher differences were observed as the relevant level decreased from 1.20 mg/kg to 0.32 mg/kg in Atlantic bonito and 0.95 mg/kg to 0.16 mg/kg in bluefin tuna, which means the smoking process reduced the excessive Pb level to an acceptable limit in these two species. In contrast, the Pb level was increased from 0.19 mg/kg to 3.20 mg/kg in sea bass. The concentrations of Pb, Cd, Hg, Cr, and Ar in commercial smoked catfish samples studied were very far below the maximum permissible limits approved by World Health Organization for grilled and smoked meat and fish, which are Pb (0.3 mg/kg); Cd (0.2 mg/kg), Hg (0.2 mg/kg), and Cr (0.3 mg/kg), and therefore the products will not constitute health risks to consumers. In three (rainbow trout, blue fin tuna and horse mackerel) of the five tested species, the health risks were found below the maximum permissible limits approved by EU (EC 2001 and the Turkish Food Codex (2011). The remaining species; in particular sea bass, Pb value levels were above the limit value of 0.03 mg/kg. Similarly, Şireli et al. [55] reported that the Pb value of smoked fish samples differed from 0.01 to 0.8 mg/kg. Anigboro et al. [56] also detected the Pb level range between 13 and 59 mg/kg in different species of smoked fish.

Except for bluefin tuna and seabass, the concentration of arsenic decreased following the smoking process; the following levels were found in rainbow trout (0.02 mg/kg), Atlantic bonito (1.18 mg/kg), bluefin tuna (2.16 mg/kg), sea bass (0.20 mg/kg) and horse mackerel (0.18 mg/kg). These findings are supported by previous research conducted by Töre et al. [51], whose results show significant differences in As levels across various species. The Cd level of rainbow trout, Atlantic bonito, and bluefin tuna were decreased after the smoking process which was also reported by Abbas et al. [57] who reported the Cd level decreased following the smoking approach in both grass carp fish and mullet fish. Except for smoked rainbow tuna, all the raw and smoked fish muscle were found to contain Cd above the maximum limit (0.05 mg/kg settled by EU, (EC 2001 and the Turkish Food Codex (2011))). The highest to lowest level of Cd in the smoked samples can be presented as sea bass > horse mackerel > Atlantic bonito > bluefin tuna > rainbow trout. The Cd level variation depending on species was also reported by Iko Afé et al. [14] who concluded that the Cd level in fish species differed from 0.002 to 19.5 mg/kg. The Cr levels in both raw and smoked fish were found above the acceptable limits by the EU commission and Turkish Food Codex. The EU has thus set the limits of Cd in the muscle meat of mackerel at 0.10 mg/kg, and other fish at 0.05 mg/kg; for Pb and Hg they are 0.30 and 0.50 mg/kg, respectively (European Commission, 2015). While the highest Cr level was found in horse mackerel among the raw materials, the highest concentration (2.07 mg/kg) was found in smoked sea bass at 3.88 mg/kg. The differences were also found in other research that reported the Cr level varies in different fish, namely mangar, trout barb, common carp, Tigris scraper and Euphrates barbell [58]. There is no permissible maximum limit for As and Cr level in the seafood in both the Turkish Food Codex 2011 and European commission EC (2001). In the proximal composition parameters, positive correlations were found between lipid content and PAH4 level (R^2^ = (r = 0.875, *p* < 0.05). No correlations were found between protein and PAH4 contents of tested fish sample (R^2^ = 0.07, *p* < 0.05)

Concentrations (mg/kg) of Zn and Fe were found to be relatively higher in both fresh and smoked samples, regardless of fish species (they ranged from 8.76 ± 0.01 to 20.20 ± 0.04 and 5.20 ± 0.02 to 18.10 ± 0.05, respectively). Bioaccumulation of these two trace elements on fish muscle at higher levels was also reported in several other studies [20]. When comparing the fresh form of all the tested fish species, while the relatively higher zinc levels were determined in rainbow tuna (20.20 mg/kg) and Atlantic bonito (17.47 mg/kg), higher iron levels were found in bluefin tuna (18.10 mg/kg) and Atlantic bonito (15.65 mg/kg). The lowest concentration of these two trace elements were determined in fresh sea bass at 8.76 mg/kg and 7.73 mg/kg for Zn and Fe, respectively. The significant dominance of iron and zinc in the different fish species was also reported by [59]. The significant differences in the trace elements (Mn, Co, Cu, Al, Ni, Se) driven by fish species and smoking process were also given in Table 4.

While cobalt concentrations were found at the trace level in both fresh and smoked fish, samples ranged between 0.02 and 0.07 mg/kg; other trace metal levels were found at the higher limits given by fish species. When compared to trace element levels in the raw form of the tested species, while the levels of trace elements can be represented as Cu > Mn > Sn > Al >Se > Ni > Co in the rainbow trout muscle, the order determined as Mn > Cu > Al > Sn >Se > Ni > Co was changed in Atlantic bonito. In the bluefin tuna and sea bass, the trace element levels were determined as follows: Mn > Al > Cu > Sn Ni > Se > Co. The trace element from highest to lowest levels were found as Mn > Cu >Al > Sn> Se > Ni > Co in raw horse mackerel. It is thought that all these mentioned variations are caused by environmental factors and feeding and sampling area differences, which was also confirmed by Bibak et al. [60] who found the variations of trace metal levels in the two species from the same gulf. In this study, the highest Cu level was found in the rainbow trout, which has also the highest protein content, and the highest level of Mn level was detected in horse mackerel which has the lowest total lipid content among the fish species. The variations in the trace metal levels among tested species might be due to the proximal differences of fish species. This result agrees with Canli and Atli, [61] who reported that it is clear that the organic compounds such as lipid and protein impact on the bioaccumulation of trace elements. Statically significant differences were also determined in the trace element levels in the tested fish samples after the smoking process. (Table 4, Figure 2B). While except Mn, all the other trace elements level decreased after the smoking process in rainbow trout; in bluefin tuna, all the trace element levels were reduced except Cu. The relatively bigger difference between fresh and smoked forms of the tested species were observed in horse mackerel. In the present work, the Al level increased from 1.71 mg/kg to 2.05 mg/kg; Mn level increased from 2.74 mg/kg to 5.02 mg/kg, Sn concentrations increased from 0.37 mg/kg to 2.07 mg/kg in horse mackerel. These results are in agreement with Abbas et al. [57] who reported that the trace element level of fish muscle differed following the smoking process. While the trace elements are essential for the metabolism, since the higher concentrations of these elements can cause toxicity, it is necessary in fish products, especially in the complex processing methods applied, such as smoking and canning.

The levels of macroelements (Mg and Ca) in the different fish muscle before and after the smoking process were determined in Table 4, Figure 2C. In terms of Mg, the highest (0.89 mg/kg) and lowest (0.45 mg/kg) levels were determined in horse mackerel and sea bass, respectively. The slight differences in the Mg concentrations were detected among smoked fish species samples. Another macro-element, the Ca levels of raw fish species differed between 1.56 and 2.13 mg/kg, which are in accordance with previous research that reported the Ca levels varied from 1.6 to 6.2 mg/kg in different species [62]. Similarly to the other elemental variation, a significant increase and decrease was observed after the smoking process. While the Ca level decreased from 2.12 to 195 in Atlantic bonito, this level decreased from 2.13 mg/kg to 1.86 mg/kg in sea bass. The Ca level increased from 1.56 mg/kg to 2.04 mg/kg in rainbow trout, from 1.75 mg/kg to 1.80 mg/kg in bluefin tuna, and from 1.89 mg/kg to 1.99 mg/kg in horse mackerel. These results are in agreement with Olukayode Amos, and Paulina, [63] who concluded that the Ca level was in the range between 2.26 and 3.13 mg/kg in Clarias gariepinus, and Synodontis budgetti, respectively. The variations of macro-elements in processed fish are mainly reasoned by brining steps [64].

### 3.5. Health Risk Assessment

Table 5 summarizes the health risk assessment of PAHs in the analyzed raw and smoke fish samples for a person (an adult, 70 kg) within their dietary daily intake DDI and the carcinogenic potencies of individual PAHs.

The DDI values (mg/day) differed depending on estimated from individual PAH concentrations in both raw and smoked form of fish species. DDI values between the tested fish species were found to be statistically significant (*p* < 0.05). DDI values of Benzo(a)anthracene (mg/day) were found to be extremely higher than other PAHs, as a result of the detected high level of this component on the fish muscle. As compared to smoked fish; DDI was determined in rainbow trout to range between 0 to 0.69, from 0.17 to 1.38 for Atlantic bonito. This value ranged between 0 and 1.04 for bluefin tuna, 0 to 0.69 for sea bass and 0.69 to 2.07 in horse mackerel. These results are in agreement with Tongo et al., who reported that the DDI for each PAHs differed in different smoked fish samples from C. gariepinus to T. zilli. The most dangerous parameter of PAHs, the benzo(a)pyrene level, did not exceed the maximum acceptable concentrations of 2.0 µg kg^−1^ as permitted by The Turkish Codex Regulation (2008) [65] in any raw or smoked sample. The carcinogenic potencies of individual PAHs (BaPteq) were also assessed by multiplying the PAH concentration in the mussel sample by the individual TEF. TEFs have been used as a useful tool for the regulation of PAH components, for instance, PAHs, which share a common mechanism of action. The carcinogenic potencies (B(A)Pteq) of the PAH compound were also determined and the values ranged from 0.000 to 0.608. Benzo(a)pyrene, known as one of the most carcinogenic PAHs, was determined to be between 0.001 and 0.062 in all tested samples. The level of benzo(a)anthracene was found relatively higher than other tested carcinogenic potencies of PAHs in the tested samples (0.066–0.608). The B(A)Pteq results obtained in this study clearly showed that carcinogenic potencies of PAHs differed depending on smoking and tested sample properties that are in agreement with previous research. The observed B(A)Pteq values were relatively lower than in previous research; Moslen et al. [66] reported the B(A)Pteq values ranged between 0.012 to 900.0 for mussel, and Erhunmwunse and Ekaye [67] determined that level differed from 3.91 to 8.47 in the smoked fish sample.

The estimated daily intakes of trace elements from the consumption of any of raw and smoked fish samples investigated are displayed in Table 6. The lowest and highest EDI values were found in smoked rainbow trout and smoked sea bass, respectively, with 0.01 and 0.18 µg/day for Cd. The tolerable daily intake of Cd is 0.35 μg/kg/day (EFSA 2011) [68]. All of the tested raw and smoked samples were below the recommended permissible limits. The EDI values ranged between 0.35 and 0.96 μg/kg/day in the smoked fish samples and the tolerable daily intake of Cr is declared as 3.3μg/kg day (WHO 2003) [69]. None of the tested samples did not exceed the tolerable daily intake limit. The lowest and highest EDI values were determined in smoked rainbow trout and bluefin tuna for As with 0.02 µg/day and 0.53 µg/day. The permissible values were set as 0.3 µg/kg/day for As [70]. Only one smoked fish sample (smoked bluefin tuna) has a daily intake value exceeding the tolerable daily intake. The daily intake for Pb differed from 0.04 to 0.79 µg/kg/day in the smoked fish samples. Only one smoked fish sample (smoked sea bass) exceeded the tolerable daily intake value (0.3 µg/kg/day). The EDI values of Hg ranged between 0.01 and 0.09 µg/kg/day and all the tested samples were below the permissible values, as 0.57 µg/kg/day. These results clearly showed that the EDI of trace element differ depending on fish species which is in agreement with Çulha et al. [71] who highlighted that the daily intake rate at the different rate in various fish species. In the risk evaluation with regard to THQ value for Cr, Pb, As, Cd and Hg in the smoked fish muscles were shown at Table 6. All the tested parameters found lower than 1, which indicates there is no concern for human health even in the EDI value exceeded groups [71]. Regarding the trace metals, the THQ of As was found to be much higher than any other element, however, its values were far below 1.

## 4. Conclusions

This study revealed the smoking process on the PAHs and elemental profile of different species within the same smoking process. The proximal composition and some important parameters, such as water holding capacity, processing wield and Ph were evaluated, which are important for the sustainability of the fish smoking market. The PAH profiles were investigated in terms of PAH4, PAH8 and total PAHS; elemental composition was deeply evaluated as were macro elements, trace elements and toxic elements. The results clearly revealed that the origin of species and proximate composition affected the process yield, pH and water holding capacity. The proximate composition has also strong impact on bioaccumulation of PAHs and trace and toxic metals at different ranges. While all the PAH levels were detected below the maximum limit in all smoked samples, some of the trace and toxic metals were detected above the maximum permission limit. The bioaccumulation of some toxic metals reduced and/or increased in different species, which means the relationship between the proximate composition and PAHs and elemental composition of fish species both fresh and smoked fillets. That means there is no positive and negative impact of smoking process on these component presences. In terms of the bioaccumulation of potentially toxic PAHs and heavy metal, horse mackerel was found to be more vulnerable than the other fish species. A health risk assessment of the PAHs and trace elements clearly showed that consumption of the tested raw and smoked fish sample may not cause any health risk.

## Figures and Tables

**Table 1:** Proximate composition in the fresh and smoked fillets of fish species.

Fish Species	Condition	Crude Protein	Total Lipid	Moisture	Ash
Rainbow trout	Raw	22.9 ± 0.12 ^c^	4.5 ± 0.11 ^a^	74.8 ± 2.32 ^c^	4.2 ± 0.14 ^c^
	Smoked	23.7 ± 0.21 ^B^	3.6 ± 0.13 ^A^	72.5 ± 1.45 ^D^	4.7 ± 0.12 ^C^
Atlantic bonito	Raw	14.3 ± 1.25 ^a^	17.8 ± 0.14 ^c^	68.2 ± 1.22 ^b^	1.8 ± 0.09 ^ab^
	Smoked	16.3 ± 1.10 ^A^	15.1 ± 1.22 ^C^	64.2 ± 2.21 ^B^	3.1 ± 0.24 ^B^
Atlantic bluefin tuna	Raw	21.9 ± 1.14 ^c^	10.7 ± 0.14 ^b^	63.9 ± 1.45 ^a^	2.1 ± 0.06 ^b^
	Smoked	21.6 ± 0.89 ^B^	9.9 ± 1.12 ^B^	62.5 ± 2.14 ^B^	1.7 ± 0.14 ^A^
Sea bass	Raw	19.8 ± 1.12 ^b^	4.2 ± 0.44 ^a^	69.2 ± 1.45 ^b^	1.3 ± 0.21 ^a^
	Smoked	23.9 ± 1.02 ^B^	3.5 ± 0.61 ^A^	68.4 ± 2.14 ^C^	2.9 ± 0.16 ^B^
Horse mackerel	Raw	15.7 ± 1.41 ^a^	25.4 ± 1.14 ^d^	53.4 ± 1.16 ^a^	1.5 ± 0.05 ^a^
	Smoked	17.2 ± 1.05 ^A^	23.9 ± 1.58 ^D^	53.5 ± 1.74 ^A^	1.7 ± 0.07 ^A^

Data are expressed as mean value ± standard deviation of triplicates. Values followed by different lower letters in the same column indicate significant differences in raw fish groups, the upper letters in the same column indicate the significant differences in smoked fish groups (*p* < 0.05).

**Table 2:** pH, WHC and yield of fresh and smoked fish fillets.

Fish Species	Condition	pH	WHC (%)	Process Yield (%)
Rainbow trout	Raw	5.8 ± 0.06 ^a^	83.71 ± 1.87 ^b^	71.4 ± 0.06 ^2^
	Smoked	5.3 ± 0.01 ^A^	87.96 ± 1.83 ^A^	
Atlantic bonito	Raw	5.8 ± 0.04 ^a^	89.54 ± 2.16 ^c^	88.5 ± 0.79 ^4^
	Smoked	5.4 ± 0.02 ^A^	92.55 ± 3.15 ^B^	
Atlantic bluefin tuna	Raw	6.8 ± 0.06 ^c^	81.45 ± 2.55 ^a^	84.6 ± 1.15 ^3^
	Smoked	6.3 ± 0.07 ^B^	88.57 ± 3.14 ^A^	
Sea bass	Raw	6.2 ± 0.04 ^b^	83.40 ± 2.60 ^b^	71.1 ± 0.95 ^1^
	Smoked	6.0 ± 0.02 ^A^	93.42 ± 5.17 ^B^	
Horse mackerel	Raw	6.4 ± 0.04 ^b^	84.61 ± 3.14 ^b^	90.2 ± 1.89 ^5^
	Smoked	6.1 ± 0.06 ^A^	91.45 ± 2.13 ^B^	

Data are expressed as mean value ± standard deviation of triplicates. Values followed by different lower letters in the same column indicate significant differences in raw fish groups, the upper letters indicate the significant differences in smoked fish groups (*p* < 0.05). The numbers indicate the differences among groups.

**Table 3:** Polycyclic aromatic hydrocarbons contents of raw and smoked fish samples (µg/kg).

	Rainbow Trout		Atlantic Bonito		Atlantic Bluefin Tuna		Sea Bass		Horse Mackerel	
	Raw	Smoked	Raw	Smoked	Raw	Smoked	Raw	Smoked	Raw	Smoked
Naphthalene	0.01 ± 0.00 ^a^	0.03 ± 0.01	0.01 ± 0.00 ^a^	0.06 ± 0.02	Nd	Nd	Nd	0.04 ± 0.02	0.02 ± 0.00 ^a^	0.04 ± 0.02
Acenaphthylene	0.01 ± 0.00 ^a^	0.02 ± 0.01 ^A^	Nd	0.04 ± 0.01 ^A^	Nd	Nd	0.03 ± 0.01 ^b^	0.03 ± 0.01 ^A^	0.01 ± 0.00 ^a^	0.08 ± 0.02 ^B^
1.2-Benzanthracene	Nd	Nd	Nd	0.02 ± 0.00 ^A^	Nd	Nd	Nd	Nd	0.01 ± 0.00	0.04 ± 0.01 ^B^
Acenaphthene	Nd	Nd	0.01 ± 0.00 ^a^	0.08 ± 0.00 ^C^	0.04 ± 0.01 ^b^	0.06 ± 0.02 ^B^	0.01 ± 0.0 ^a^	0.02 ± 0.00 ^A^	0.02 ± 0.0 ^a^	0.06 ± 0.01 ^B^
Benzo[k]fluoranthene	Nd	Nd	0.01 ± 0.00 ^a^	0.01 ± 0.00 ^A^	0.02 ± 0.01 ^b^	0.03 ± 0.01 ^B^	0.02 ± 0.00 ^b^	0.02 ± 0.01 ^B^	Nd	0.10 ± 0.0 ^C^
Phenanthrene	Nd	Nd	0.01 ± 0.00 ^a^	0.06 ± 0.02 ^B^	0.04 ± 0.01 ^b^	0.04 ± 0.01 ^A^	0.02 ± 0.01 ^a^	0.01 ± 0.00 ^A^	Nd	0.12 ± 0.0 ^B^
Anthracene	0.02 ± 0.01 ^a^	0.04 ± 0.02 ^B^	0.03 ± 0.01 ^b^	0.04 ± 0.00 ^B^	0.01 ± 0.00 ^a^	0.01 ± 0.00 ^A^	Nd	Nd	0.06 ± 0.02 ^c^	0.09 ± 0.03 ^C^
Fluoranthene	0.01 ± 0.00 ^a^	0.01 ± 0.01 ^A^	0.01 ± 0.00 ^a^	0.04 ± 0.02 ^B^	0.01 ± 0.00 ^a^	0.02 ± 0.01 ^A^	0.02 ± 0.01 ^b^	0.03 ± 0.01 ^B^	0.06 ± 0.01 ^c^	0.08 ± 0.02 ^C^
Pyrene	Nd	Nd	0.01 ± 0.00 ^a^	0.06 ± 0.04 ^B^	0.02 ± 0.0 ^b^	0.04 ± 0.0 ^A^	0.02 ± 0.01 ^b^	0.02 ± 0.01 ^A^	0.02 ± 0.01 ^b^	0.05 ± 0.01 ^B^
Benzo[ghi]perylene	0.01 ± 0.00 ^a^	0.02 ± 0.01	0.02 ± 0.00 ^b^	0.08 ± 0.02 ^B^	Nd	Nd	0.01 ± 0.00 ^a^	0.02 ± 0.00 ^A^	0.04 ± 0.02 ^b^	0.12 ± 0.02 ^C^
Indeno(1.2.3-cd)pyrene	0.01 ± 0.0 ^a^	0.02 ± 0.0 ^A^	Nd	0.01 ± 0.0 ^A^	0.02 ± 0.01 ^b^	0.02 ± 0.00 ^A^	0.01 ± 0.00 ^a^	0.02 ± 0.01 ^A^	0.04 ± 0.02 ^b^	0.08 ± 0.0 ^B^
Dibenzo(a.h)anthracene	Nd	Nd	Nd	0.02 ± 0.01 ^A^	Nd	Nd	0.01 ± 0.00 ^a^	0.01 ± 0.00 ^A^	0.02 ± 0.01 ^a^	0.07 ± 0.02 ^B^
Benzo(b)fluoranthene	0.03 ± 0.01 ^b^	0.03 ± 0.01 ^A^	0.01 ± 0.00 ^a^	0.01 ± 0.00 ^A^	0.02 ± 0.01 ^b^	0.02 ± 0.01 ^A^	0.01 ± 0.00 ^a^	0.02 ± 0.01 ^A^	0.02 ± 0.00 ^b^	1.57 ± 0.42 ^B^
Benzo(a)anthracene	0.79 ± 0.13 ^ab^	1.13 ± 0.15 ^A^	0.80 ± 0.12 ^b^	6.08 ± 1.23 ^C^	0.66 ± 0.14 ^a^	1.26 ± 0.13 ^A^	1.08 ± 0.19 ^c^	1.13 ± 0.21 ^A^	1.19 ± 0.13 ^c^	2.56 ± 0.78 ^B^
Benzo(a)pyrene	0.01 ± 0.00 ^a^	0.01 ± 0.00 ^A^	0.02 ± 0.0 ^a^	0.01 ± 0.0 ^A^	0.01 ± 0.00 ^a^	0.02 ± 0.00 ^A^	0.01 ± 0.00 ^a^	0.02 ± 0.0 ^A^	0.02 ± 0.00 ^a^	0.62 ± 0.05 ^B^
Chrysene	0.02 ± 0.01 ^a^	0.04 ± 0.01 ^B^	Nd	1.42 ± 0.24 ^D^	0.01 ± 0.00 ^a^	0.01 ± 0.01 ^A^	0.03 ± 0.01 ^b^	0.06 ± 0.02 ^B^	0.04 ± 0.02 ^b^	1.06 ± 0.14 ^C^

Data are expressed as mean value ± standard deviation of triplicates. Values followed by different lower letters in the same line indicate significant differences in raw fish groups, the upper letters in the same line indicate the significant differences in smoked fish groups. (*p* < 0.05).

**Table 4:** Elemental profile of raw and smoked fish samples (mg/kg).

	Rainbow Trout		Atlantic Bonito		Atlantic Bluefin Tuna		Sea Bass		Horse Mackerel	
	Raw	Smoked	Raw	Smoked	Raw	Smoked	Raw	Smoked	Raw	Smoked
Al	1.65 ± 0.03 ^a^	1.63 ± 0.01 ^AB^	1.83 ± 0.12 ^ab^	2.17 ± 0.15 ^C^	3.03 ± 0.02 ^c^	1.24 ± 0.04 ^A^	2.74 ± 0.10 ^b^	2.76 ± 0.11 ^D^	1.71 ± 0.21 ^a^	2.05 ± 0.05 ^B^
Cr	1.69 ± 0.07 ^ab^	1.72 ± 0.02 ^B^	1.50 ± 0.05 ^a^	1.64 ± 0.08 ^B^	1.64 ± 0.04 ^ab^	1.42 ± 0.06 ^AB^	1.51 ± 0.14 ^a^	3.88 ± 0.15 ^C^	2.07 ± 0.13 ^b^	1.53 ± 0.06 ^A^
Mn	2.35 ± 0.15 ^a^	2.61 ± 0.21 ^B^	2.96 ± 0.14 ^b^	2.43 ± 0.12 ^B^	3.59 ± 0.45 ^c^	1.56 ± 0.22 ^A^	3.27 ± 0.03 ^b^	4.55 ± 0.13 ^C^	2.74 ± 0.04 ^ab^	5.02 ± 0.1 ^D^
Co	0.07 ± 0.01 ^b^	0.02 ± 0.01 ^A^	0.05 ± 0.01 ^ab^	0.06 ± 0.02 ^B^	0.03 ± 0.01 ^a^	0.02 ± 0.01 ^A^	0.02 ± 0.00 ^a^	0.04 ± 0.01 ^AB^	0.03 ± 0.01 ^a^	0.02 ± 0.01 ^A^
Ni	0.23 ± 0.02 ^ab^	0.12 ± 0.01 ^A^	0.22 ± 0.03 ^ab^	0.35 ± 0.05 ^C^	0.38 ± 0.12 ^b^	0.12 ± 0.02 ^A^	0.16 ± 0.04 ^a^	0.25 ± 0.05 ^B^	0.22 ± 0.02 ^ab^	0.21 ± 0.03 ^B^
Cu	3.62 ± 0.18 ^c^	2.03 ± 0.11 ^A^	2.11 ± 0.16 ^b^	2.61 ± 0.17 ^B^	2.19 ± 0.11 ^b^	2.23 ± 0.14 ^A^	1.48 ± 0.12 ^a^	2.31 ± 0.05 ^A^	2.61 ± 0.16 ^bc^	2.60 ± 0.25 ^B^
Zn	20.20 ± 1.25 ^e^	8.81 ± 0.15 ^A^	17.47 ± 1.22 ^d^	12.53 ± 0.11 ^B^	14.57 ± 1.15 ^c^	11.43 ± 1.12 ^B^	8.76 ± 0.20 ^a^	15.62 ± 0.19 ^C^	11.77 ± 0.15 ^b^	16.36 ± 1.24 ^D^
As	1.33 ± 0.15 ^b^	0.09 ± 0.01 ^A^	1.21 ± 0.16 ^b^	1.18 ± 0.25 ^C^	0.13 ± 0.02 ^a^	2.16 ± 0.45 ^D^	0.14 ± 0.02 ^a^	0.20 ± 0.02 ^B^	0.20 ± 0.04 ^a^	0.18 ± 0.02 ^B^
Se	0.43 ± 0.06 ^b^	0.22 ± 0.03 ^A^	0.35 ± 0.02 ^a^	0.36 ± 0.04 ^C^	0.34 ± 0.16 ^a^	0.31 ± 0.08 ^B^	0.31 ± 0.04 ^a^	0.30 ± 0.04 ^AB^	0.29 ± 0.04 ^a^	0.32 ± 0.04 ^B^
Fe	14.08 ± 1.04 ^b^	5.20 ± 0.41 ^A^	15.65 ± 1.05 ^b^	11.67 ± 0.04 ^B^	18.10 ± 1.52 ^c^	10.29 ± 1.05 ^B^	7.73 ± 1.01 ^a^	14.65 ± 0.05 ^C^	13.77 ± 0.16 ^b^	12.58 ± 1.19 ^C^
Cd	0.06 ± 0.01 ^a^	0.05 ± 0.01 ^A^	0.31 ± 0.04 ^c^	0.12 ± 0.02 ^B^	0.14 ± 0.02 ^b^	0.07 ± 0.01 ^A^	0.06 ± 0.02 ^a^	0.74 ± 0.02 ^C^	0.08 ± 0.02 ^a^	0.15 ± 0.03 ^B^
Sn	2.27 ± 0.04 ^c^	0.06 ± 0.01 ^A^	1.15 ± 0.07 ^b^	2.63 ± 0.13 ^C^	0.45 ± 0.04 ^ab^	0.20 ± 0.04 ^B^	0.44 ± 0.04 ^ab^	0.36 ± 0.04 ^B^	0.37 ± 0.01 ^a^	2.07 ± 0.04 ^C^
Mg	0.73 ± 0.03 ^b^	0.32 ± 0.02 ^A^	0.59 ± 0.04 ^ab^	0.80 ± 0.04 ^C^	0.74 ± 0.06 ^b^	0.61 ± 0.06 ^C^	0.45 ± 0.05	0.45 ± 0.03 ^B^	0.89 ± 0.03 ^b^	0.64 ± 0.02 ^B^
Hg	0.29 ± 0.01 ^b^	0.07 ± 0.02 ^A^	0.39 ± 0.03 ^b^	0.03 ± 0.01 ^A^	0.28 ± 0.02 ^b^	0.12 ± 0.02 ^B^	0.37 ± 0.02 ^b^	0.35 ± 0.01 ^C^	0.02 ± 0.01 ^a^	0.21 ± 0.03 ^B^
Pb	0.31 ± 0.03 ^b^	0.26 ± 0.06 ^B^	1.20 ± 0.14 ^d^	0.31 ± 0.09 ^B^	0.95 ± 0.05 ^c^	0.16 ± 0.04 ^A^	0.19 ± 0.03 ^a^	3.2 ± 0.19 ^C^	0.34 ± 0.12 ^b^	0.28 ± 0.04 ^B^
Ca	1.56 ± 0.24 ^a^	2.04 ± 0.13 ^C^	2.12 ± 0.21 ^c^	1.95 ± 0.24 ^B^	1.75 ± 0.15 ^ab^	1.80 ± 0.23 ^A^	2.13 ± 0.15 ^c^	1.86 ± 0.15 ^A^	1.89 ± 0.24 ^b^	1.99 ± 0.05 ^B^

Data are expressed as mean value ± standard deviation of triplicates. Values followed by different lower letters in the same line indicate significant differences in raw fish groups, the upper letters in the same line indicate the significant differences in smoked fish groups. (*p* < 0.05).

**Table 5:** Health Risk assessment of PAHs.

	DDI										PAHs (B(A)Pteq									
	Rainbow Trout		Atlantic Bonito		Atlantic Bluefin Tuna		Sea Bass		Horse Mackerel		Rainbow Trout		Atlantic Bonito		Atlantic Bluefin Tuna		Sea Bass		Horse Mackerel	
	Raw	Smoked	Raw	Smoked	Raw	Smoked	Raw	Smoked	Raw	Smoked	Raw	Smoked	Raw	Smoked	Raw	Smoked	Raw	Smoked	Raw	Smoked
NaP	0.17	0.52	0.17	1.04	0.00	0.00	0.00	0.69	0.35	0.69	0.00	0.00	0.00	0.00	0.00	0.00	0.00	0.00	0.00	0.00
AcPY	0.17	0.35	0.00	0.69	0.00	0.00	0.52	0.52	0.17	1.38	0.00	0.00	0.00	0.00	0.00	0.00	0.00	0.00	0.00	0.00
BaA	0.00	0.00	0.00	0.35	0.00	0.00	0.00	0.00	0.17	0.69	0.00	0.00	0.00	0.00	0.00	0.00	0.00	0.00	0.00	0.00
Ant	0.00	0.00	0.17	1.38	0.69	1.04	0.00	0.35	0.35	1.04	0.00	0.00	0.00	0.00	0.00	0.00	0.00	0.00	0.00	0.00
BkFL	0.00	0.00	0.17	0.17	0.35	0.52	0.35	0.35	0.00	1.73	0.00	0.00	0.00	0.00	0.00	0.00	0.00	0.00	0.00	0.00
Phe	0.00	0.00	0.17	1.04	0.69	0.69	0.35	0.17	0.00	2.07	0.00	0.00	0.00	0.00	0.00	0.00	0.00	0.00	0.00	0.00
Ant	0.35	0.69	0.52	0.69	0.17	0.17	0.00	0.00	1.04	1.56	0.00	0.00	0.00	0.00	0.00	0.00	0.00	0.00	0.00	0.00
FL	0.17	0.17	0.17	0.69	0.17	0.35	0.35	0.52	1.04	1.38	0.00	0.00	0.00	0.00	0.00	0.00	0.00	0.00	0.00	0.00
Pyr	0.00	0.00	0.17	1.04	0.35	0.69	0.35	0.35	0.35	0.86	0.00	0.00	0.00	0.00	0.00	0.00	0.00	0.00	0.00	0.00
BP	0.17	0.35	0.35	1.38	0.00	0.00	0.17	0.35	0.69	2.07	0.00	0.00	0.00	0.00	0.00	0.00	0.00	0.00	0.00	0.00
Ind	0.17	0.35	0.00	0.17	0.35	0.35	0.17	0.35	0.69	1.38	0.00	0.00	0.00	0.00	0.00	0.00	0.00	0.00	0.00	0.01
DBA	0.00	0.00	0.00	0.35	0.00	0.00	0.17	0.17	0.35	1.21	0.00	0.00	0.00	0.00	0.00	0.00	0.00	0.00	0.00	0.00
BbFL	0.52	0.52	0.17	0.17	0.35	0.35	0.17	0.35	0.35	27.15	0.00	0.00	0.00	0.00	0.00	0.00	0.00	0.00	0.00	0.00
BaA	13.66	19.54	13.83	105.12	11.41	21.79	18.67	19.54	20.58	44.26	3.95	5.65	4.00	30.40	3.30	6.30	5.40	5.65	5.95	12.80
BaP	0.17	0.17	0.35	0.17	0.17	0.35	0.17	0.35	0.35	10.72	0.00	0.00	0.00	0.00	0.00	0.00	0.00	0.00	0.00	0.00
Chr	14.70	20.92	14.35	130.02	12.10	22.65	19.54	21.27	21.96	100.45	0.00	0.00	0.00	0.01	0.00	0.00	0.00	0.00	0.00	0.01
PAH4	15.22	22.13	15.04	132.61	12.79	23.34	20.23	22.13	24.38	97.69	0.00	0.00	0.00	0.01	0.00	0.00	0.00	0.00	0.00	0.00
Pah8	44.61	63.63	44.78	373.12	38.90	71.58	60.17	65.53	70.20	298.25	0.00	0.00	0.00	0.01	0.00	0.00	0.00	0.00	0.00	0.01
Total PAHs	0.17	0.52	0.17	1.04	0.00	0.00	0.00	0.69	0.35	0.69	0.00	0.00	0.00	0.01	0.00	0.00	0.00	0.00	0.00	0.01

**Table 6:** Health Risk assessment of Heavy metals.

		EDI					THQ				
Fish Species	Condition	Cr	As	Cd	Hg	Pb	Cr	As	Cd	Hg	Pb
Rainbow trout	Raw	0.42	0.33	0.01	0.07	0.08	0.06	0.44	0.06	0.10	0.01
	Smoked	0.42	0.02	0.01	0.02	0.06	0.06	0.03	0.05	0.02	0.01
Atlantic bonito	Raw	0.37	0.30	0.08	0.10	0.30	0.05	0.40	0.31	0.13	0.03
	Smoked	0.41	0.29	0.03	0.01	0.08	0.05	0.39	0.12	0.01	0.01
Atlantic bluefin tuna	Raw	0.41	0.03	0.03	0.07	0.23	0.05	0.04	0.14	0.09	0.02
	Smoked	0.35	0.53	0.02	0.03	0.04	0.05	0.71	0.07	0.04	0.00
Sea bass	Raw	0.37	0.03	0.01	0.09	0.05	0.05	0.05	0.06	0.12	0.00
	Smoked	0.96	0.05	0.18	0.09	0.79	0.13	0.07	0.73	0.12	0.08
Horse mackerel	Raw	0.51	0.05	0.02	0.00	0.08	0.07	0.07	0.08	0.01	0.01
	Smoked	0.38	0.04	0.04	0.05	0.07	0.05	0.06	0.15	0.07	0.01

## Data Availability

The data presented in this study are available in this manuscript.

## Supplementary Materials

The following supporting information can be downloaded at: https://www.mdpi.com/article/10.3390/molecules27207015/s1. Table S1: LOD and R^2^ values of PAHs analysed in this study; Table S2. LOD and R2 values of elements analysed in this study.

## Sample Availability

Samples of the compounds are not available from the author.
