# Supplementary material for: Investigations of the Polycyclic Aromatic Hydrocarbon and Elemental Profile of Smoked Fish
Source: Molecules. 2022 Oct 18;27(20):7015. doi: 10.3390/molecules27207015 (PMC9608441; doi:10.3390/molecules27207015)
Supplement: Supplementary file 1 [file molecules-27-07015-s001.zip › molecules-1920558-supplementary.pdf]

Table S1. LOD and R^2 values of PAHs analysed in this study

PAHs compound	LOD value (μg/ml)	R^2
Naphthalene	0.0001 mg/kg	0.9995
Acenaphthylene	0.0001 mg/kg	0.9991
1,2-Benzanthracene	0.0001 mg/kg	0.9995
Acenaphthene	0.0001 mg/kg	0.9994
Benzo[k]fluoranthene	0.0001 mg/kg	0.9994
Phenanthrene	0.0001 mg/kg	0.9995
Anthracene	0.0001 mg/kg	0.9995
Fluoranthene	0.0001 mg/kg	0.9994
Pyrene	0.0001 mg/kg	0.9998
Benzo[ghi]perylene	0.001 mg/kg	0.999
Indeno(1,2,3-cd)pyrene	0.001 mg/kg	0.9997
Dibenzo(a,h)anthracene	0.001 mg/kg	0.9998
Benzo(b)fluoranthene	0.001 mg/kg	0.9976
Benzo(a)anthracene	0.001 mg/kg	0.9997
Benzo(a)pyrene	0.001 mg/kg	0.9994
Chrysene	0.001 mg/kg	0.9985

Table S2. LOD and R^2 values of elemnts analysed in this study

Element	LOD value (µg/ml)	R^2
Al	0.2113	0.9994
Cr	0.0319	0.9989
Mn	0.0227	0.9991
Fe	11.742	0.9986
Co	0.0051	0.9998
Ni	0.1874	0.9995
Cu	0.1377	0.9995
Zn	0.1512	0.9994
As	0.0241	0.9998
Cd	0.0238	0.9998
Sn	0.0482	0.9994
Se	0.0043	0.9996
Hg	0.0488	0.9971
Pb	0.0381	0.9996
Mg	0.3274	0.9989
Ca	4.388	0.9987
